# Long-Term Isolation Elicits Depression and Anxiety-Related Behaviors by Reducing Oxytocin-Induced GABAergic Transmission in Central Amygdala

**DOI:** 10.3389/fnmol.2018.00246

**Published:** 2018-08-14

**Authors:** Rafael T. Han, Young-Beom Kim, Eui-Ho Park, Jin Yong Kim, Changhyeon Ryu, Hye Y. Kim, JaeHee Lee, Kisoo Pahk, Cui Shanyu, Hyun Kim, Seung K. Back, Hee J. Kim, Yang In Kim, Heung S. Na

**Affiliations:** ^1^Neuroscience Research Institute and Department of Physiology, Korea University College of Medicine, Seoul, South Korea; ^2^Department of Anatomy, Korea University College of Medicine, Seoul, South Korea; ^3^Neuroscience Research Institute and Department of Physiology, College of Medicine, Seoul National University, Seoul, South Korea; ^4^Department of Neuroscience, Korea University College of Medicine, Seoul, South Korea; ^5^Department of Pharmaceutics and Biotechnology, College of Medical Engineering, Konyang University, Chungnam, South Korea; ^6^Division of Biological Science and Technology, Science and Technology College, Yonsei University, Wonju, South Korea

**Keywords:** oxytocin, inhibitory synaptic transmission, central amygdala (CeA), gamma-aminobutyric acid, isolation, depression and anxiety disorders

## Abstract

Isolation stress is a major risk factor for neuropsychiatric disorders such as depressive and anxiety disorders. However, the molecular mechanisms underlying isolation-induced neuropsychiatric disorders remain elusive. In the present study, we investigated the subcellular mechanisms by which long-term isolation elicits depression and anxiety-related behaviors in mice. First, we found that long-term isolation induced depression-related behaviors in the forced swimming test (FST) and the sucrose preference test, as well as anxiety-related behaviors in the elevated zero maze test (EZMT) and the open field test. Next, we showed that intracentral amygdala (CeA) injection of oxytocin (OXT), but not intracerebroventricular injection, attenuated isolation-induced depression and anxiety-related behaviors via oxytocin receptor (OXTR), not vasopressin-1a receptor (V1aR), in the FST and EZMT, respectively. Quantitative real-time polymerase chain reaction analysis revealed that after 5 weeks of isolation, mRNA transcription of OXTR in the CeA, but not that of V1aR, significantly decreased, whereas OXT and vasopressin mRNA transcription in the paraventricular nucleus of hypothalamus did not change significantly. Whole-cell patch clamping of acute brain slices demonstrated that the frequency of miniature inhibitory postsynaptic currents (mIPSCs) in CeA neurons, but not their amplitude, was lower in isolated mice than in group-housed mice. Notably, OXT treatment increased the mIPSC frequency in the CeA neurons, but to a lesser extent in the case of isolated mice than in that of group-housed mice via OXTR. Taken together, our findings suggest that long-term isolation down-regulates OXTR mRNA transcription and diminishes OXT-induced inhibitory synaptic transmission in the CeA and may contribute to the development of depression and anxiety-related behaviors in isolated mice through the enhancement of CeA activity.

## Introduction

Depressive and anxiety disorders are among the most prevalent neuropsychiatric disorders (Fava et al., 2000). Despite their distinct difference in symptoms, both disorders frequently coexist in one patient (Ballenger, 1999; Ionescu et al., 2013) and share common pathophysiology and risk factors, including abnormal activity in the amygdala and social stressors, respectively (England and Sim, 2009; Grav et al., 2012; Wang et al., 2014). For example, hyperactivity in the amygdala has not only been characterized in anxiety disorder but is also implicated in depressive disorder (Shin and Liberzon, 2010; Yang et al., 2010). Abnormal processing of emotion and aberrant neural circuitry of the amygdala were implicated in patients with depressive disorder and the animal model of depression (Sheline et al., 2001; Roberson-Nay et al., 2006; Mottolese et al., 2014). In addition to the dysregulated activity of the amygdala, social stress is also a common risk factor of depressive and anxiety disorders. Social support was reported to moderate the effects of stress on depressive disorder. Conversely, social isolation is a major risk factor for depressive and anxiety disorders (Wang et al., 2014).

Oxytocin (OXT) is a neurohormone synthesized in the hypothalamus. OXT-expressing neurons project directly to other brain areas such as the amygdala (Huber et al., 2005; Knobloch et al., 2012; Boccia et al., 2013). OXT has attracted attention for its role in mediating social stress and modulating the activity of the amygdala (Huber et al., 2005; Viviani et al., 2011; Olff et al., 2013). OXT was reported to mediate isolation-induced behavioral changes and elevate social interactions in experimental animals and human studies (Han et al., 2016b; Yatawara et al., 2016). In addition, recent studies have implicated the potential roles of OXT in anxiolysis and shown therapeutic benefits of OXT for subsets of patients with depressive disorders (Slattery and Neumann, 2010b). Furthermore, oxytocinergic circuits from the hypothalamus to the amygdala have been shown to modulate fear responses (Altemus et al., 2001; Viviani et al., 2011; Knobloch et al., 2012). However, in spite of the close correlation between OXT as a mediator of social stress and neuropsychiatric disorders such as depressive and anxiety disorders, the neural mechanisms underlying the effects of social stress on the oxytocinergic circuits and the hyperactivity in the amygdala with regards to depressive and anxiety disorders still remain an unanswered question.

In the present study, we investigated the neuronal mechanisms by which long-term isolation elicits depressive and anxiety-related behaviors. First, we examined whether long-term isolation increased depression and anxiety-related behaviors in mice and whether administration of OXT into the amygdala would ameliorate the isolation-induced depression and anxiety-related behaviors. Next, we explored the molecular and functional changes in the oxytocinergic circuits of the amygdala after long-term isolation.

## Materials and Methods

### Animals

C57BL/6N mice (Orient Bio, Korea) were used in this study. Since the effect of oxytocin on stress-related behaviors and hormone secretion varies during the menstrual cycle (Altemus et al., 2001; Kotwica et al., 2004), only male mice were used. All experiments were approved by the Institutional Animal Care and Use Committee at Korea University College of Medicine (KOREA-2016-0203). Animals were housed under a 12-h light/dark cycle. Food and water were available *ad libitum*. Mice were either group housed or single housed in standard cages until the end of experimentation.

### Isolation

Mice were subjected to isolation at the age of 6 weeks after living with same-sex siblings in a standard cage since weaning. Isolation involved removing same-sex siblings from the standard cage and placing an isolated mouse into an identically sized standard cage. The isolated mice were subjected to 5 weeks of continuous isolation.

### Stereotaxic Injections

Using previously published procedures (Kim et al., 2011), drugs were injected with the use of a stereotaxic frame (Model 900, Kopf Instrument, United States) with ear bars. Coordinates according to Paxinos and Franklin (2004) were as follows (in mm, from bregma): CeA: A/P (rostrocaudal), −1.3; M/L (mediolateral), ±2.7; D/V (dorsoventral), −4.8 and lateral ventricle: A/P, −0.2; M/L, ±1.0; D/V, −2.3. Ketamine/xylazine (100 mg/kg and 10 mg/kg, respectively; i.p.) were used for anesthesia. Guide cannulas (26G, RWD, United States) were inserted through small holes drilled into the skull. A separate hole was drilled into the opposite position of the cannula insertion site on the skull. A tiny screw was inserted into the hole. Dental cement was applied to the skull for fixation of the guide cannulas. One week post-surgery, an internal cannula (30G, RWD, United States) connected to a polyethylene microtube was inserted through the guide cannula and secured in place with a fixing screw (RWD, United States). The polyethylene microtube was connected to a 10-μl gastight Hamilton syringe. Solutions were infused using the Hamilton syringe (1 μl/min) in freely moving mice. After the behavioral tests, mice were sacrificed to verify cannula placements. Animals with misplaced cannulas (17/132) were excluded from the analysis.

### Drugs

Oxytocin was purchased from Sigma–Aldrich (United States). desGly-NH_2_-d(CH_2_)_5_[D-Tyr^2^,Thr^4^]OVT (OXT receptor antagonist, OXTRA) and d(CH_2_)_5_[Tyr(Me)^2^]AVP (vasopressin-1a receptor antagonist, V1aRA) were generously donated by Dr. Manning (The University of Toledo College of Medicine and Life Sciences, Maurice Manning) (Manning et al., 2012). All drugs were diluted to the final working concentrations from stock solutions on the day of experimentation. The solutions used in behavioral tests were dissolved in normal saline with final volumes adjusted to 2 μl. For electrophysiological experiments, OXT and OXTRA were dissolved in ACSF solution at 0.3 and 1 μM, respectively (Huber et al., 2005; Tang et al., 2014).

### Behavioral Tests

Behavioral tests were performed as previously described (Kim et al., 2017). Since repeated tests affected behavioral test results, separate cohorts of mice completed each behavioral test. All the behavioral tests were begun 2 h after the light cycle had started. During experimentation, each mouse was concealed from the other mice. The forced swimming test (FST) and the sucrose preference test (SPT) were performed to measure depression-related behaviors. The open field test (OFT) and the elevated zero maze test (EZMT) were performed to measure anxiety-related behaviors.

### The Forced Swimming Test

The mice were forced to swim for 15 min in a cylindrical glass tank (13-cm diameter) filled to a depth of 20 cm with tap water at a temperature of 22–25°C. The next day, the mice were subjected to a 6-min FST in the same tank and recorded using a video camera. The immobility time was analyzed using ANY-maze (Stoelting Co., United States).

### The Sucrose Preference Test (SPT)

Two bottles were made available per cage, each containing either 200 ml of 1% sucrose (w/v) or 200 ml of tap water. After 12 h, the position of each bottle was switched for another 12 h. Preference (sucrose consumption ratio) was measured after 24 h as follows: (sucrose consumption)/(sucrose consumption + water consumption). Animals were returned to their home cages post-test.

### The Open Field Test

The apparatus consisted of a gray arena (45 × 45 × 40 cm). A mouse was placed at the corner of the floor and the time each mouse spent in the center (center square area 22.5 cm × 22.5 cm), the number of center visits, and the total movement distance were measured using infrared recording for 30 min. Between trials, the arena was cleaned with 70% ethanol.

### The Elevated Zero Maze Test

The maze comprised gray acrylic in a circular track 6-cm wide, 40 cm in diameter, and elevated 65 cm from the floor. The maze was divided into four quadrants of equal length containing two opposing open quadrants with clear acrylic curbs (1-cm high) and two opposing closed quadrants with gray acrylic walls (11-cm high). Animals were placed in the center of a closed quadrant and allowed to explore for 5 min. The percentage of time spent in the open quadrants and the number of head dips were recorded with an overhead video camera. Between trials, the maze was cleaned with 70% ethanol.

Experimenters were blinded to the drug treatment and all behavioral tests were performed after 20 min of drug administration. The drug concentrations used were determined based on previous studies (Neumann and Slattery, 2016).

### Electrophysiology

#### Slice Preparation

Brain slices were prepared as previously described (Kim et al., 2013). Briefly, coronal slices containing the CeA (300 μm) were obtained from 11- to 12-week-old mice using a vibrating microtome (Model 7000smz-2, Campden Instrument, United Kingdom) after isoflurane anesthesia and decapitation. The slices were cut with ice-cold artificial cerebrospinal fluid (ACSF) solution containing the following (in mM): 124 NaCl, 1.3 MgSO_4_, 3 KCl, 1.25 NaH_2_PO_4_, 26 NaHCO_3_, 2.4 CaCl_2_, and 10 glucose with 95% O_2_ and 5% CO_2_. For recovery, slices were incubated at 35.5°C for 60 min. All recordings were obtained within 8 h of recovery.

#### Intracellular Recordings

As previously described (Kim et al., 2011; Han et al., 2018), current- or voltage-clamp recordings were obtained from neurons in CeA-containing slices equilibrated for 1–12 h in the recording chamber. Micropipettes (Sutter Instruments, United States; tip diameter, 1.5–2.0 μm; 3–6 MΩ) pulled from borosilicate tubing (P-97; Sutter Instruments, United States) and filled with a KCl-containing internal solution (in mM: 120 KCl, 10 HEPES, 1 CaCl_2_, 2 MgCl_2_, 11 EGTA, 2 K_2_ATP, and adjusted at pH 7.2–7.3 using KOH) were used for whole-cell recordings, which were performed at 35–36°C. The signals from neurons amplified by Axoclamp-2B amplifier (bandwidth filter of 10 kHz for current-clamp recordings and 1 kHz for voltage-clamp recordings) were digitized and sampled at 50-μs intervals (Digidata 1320, pClamp 8.0; Molecular Devices, United States).

### Miniature Inhibitory Post Synaptic Current (mIPSC) Analysis

As previously described (Ryu et al., 2017), mIPSCs were recorded from neurons in the medial part of the CeA at a −80-mV holding potential in ACSF solution with 0.5-μM tetrodotoxin (TTX), 100-μM 2-amino-5-phosphonovaleric acid (AP5), and 20-μM 6,7-dinitroquinoxaline-2,3-dione (DNQX) 5 min after whole-cell patching. The mIPSC data were analyzed using Mini Analysis (Synaptosoft, United States). The ratio of changes in mIPSC frequency or amplitude was calculated as follows: mIPSC frequency (or amplitude) after OXT treatment/mIPSC frequency (or amplitude) before OXT treatment. Recordings were excluded from analysis if the series resistance varied by more than 20%.

### Quantitative Real-Time Polymerase Chain Reaction (RT-PCR)

The CeA- and paraventricular nucleus of hypothalamus- (PVN-) containing brain slices (300 μm) were obtained using Vibroslice (Model ROMA752, Campden Instruments, United Kingdom). The CeA or PVN was identified using a microscope and punched-out using 1-mm disposable punch biopsies. As previously described (Han et al., 2016a), the collected tissue was homogenized. RNA was extracted from the homogenized tissue using TRIzol Reagent (Life Technologies, United States), reverse-transcribed using M-MLV reverse transcriptase (Invitrogen, United States), and amplified by quantitative PCR with LightCycler 96 (Roche Diagnostics, Switzerland) and GoTaq qPCR Master Mix (Promega, United States), respectively, according to the manufacturer’s instructions. Primers are shown in **Table 1**.

**Table 1:** qPCR Primers.

Target gene	Forward primer (5′ - 3′)	Reverse primer (5′ - 3′)	GenBank accession
GAPDH	AAGGTCATCCCAGAGCRTGAA	CTGCTTCACCACCTTCTTGA	GU214026.1
Oxytocin receptor	GCACGGGTCAGTAGTGTCAA	AAGCTTCTTTGGGCGCATTG	NM_001081147.1
Oxytocin	AGGAGAACTACCTGCCTTCG	GGTATTCCCAGAAAGTGGGCT	NM_011025.4
Vasopressin-la receptor	GTCCGAGGGAAGACAGCATC	GATCTTGGCACGGGAAATGC	NM_016847.2
Vasopressin	TACGCTCTCCGCTTGTTTCC	AAAACCGTCGTGGCACTC	NM_009732.2

### Statistical Analysis

**FIGURE 1:**
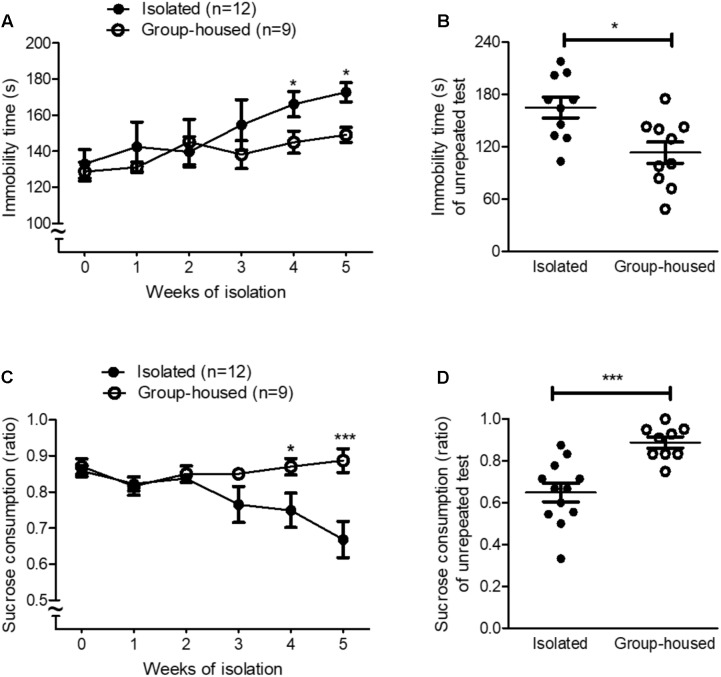
Isolated mice showed depression-related behaviors in the FST and the SPT. **(A)** Chronological changes in the immobility time of isolated and group-housed mice in the FST during the 5-week period. **(B)** Comparison of the immobility time between isolated (*n* = 10) and group-housed mice (*n* = 10) in the FST after the 5-week isolation. **(C)** Chronological changes in the sucrose consumption ratio of isolated and group-housed mice in the SPT during the 5-week period. **(D)** Comparison of the sucrose consumption ratio between the isolated (*n* = 12) and group-housed mice (*n* = 10) in the SPT after the 5-week isolation. ^∗^*p* < 0.05, ns; not significant, repeated measures two-way ANOVA followed by *post hoc* Bonferroni’s test in **(A,C)** and unpaired student’s *t*-test in **(B,D)**. Error bars represent SEM. FST; forced swimming test; SPT; sucrose preference test.

**FIGURE 2:**
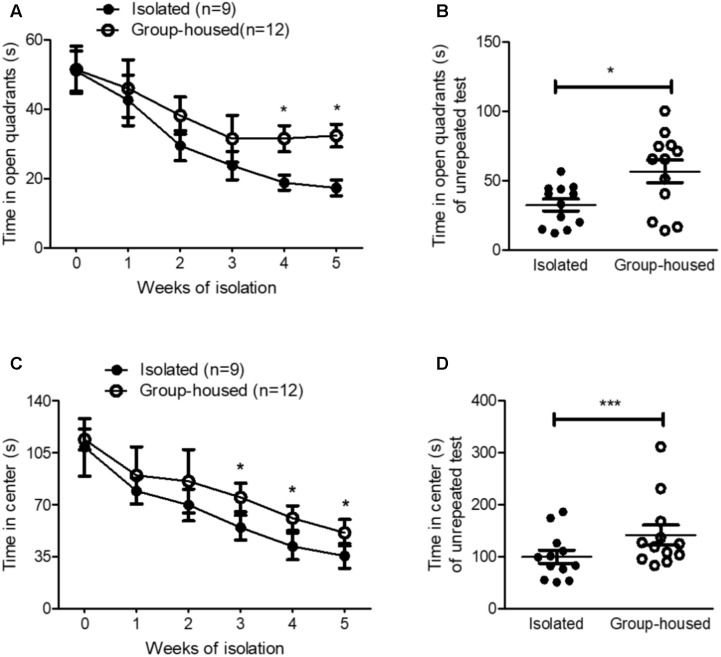
Isolated mice exhibited anxiety-related behaviors in the OFT and the EZMT. **(A)** Chronological changes in time spent in open quadrants by isolated and group-housed mice in the EZMT during the 5-week period. **(B)** Comparison of the time spent in open quadrants between isolated (*n* = 12) and group-housed (*n* = 12) mice after the 5-week isolation in the EZMT. **(C)** Chronological changes in the time spent in the center between the isolated and group-housed mice in the OFT during the 5-week period. **(D)** Comparison of the time spent in the center between the isolated (*n* = 12) and group-housed mice (*n* = 12) after the 5-week isolation in the OFT. ^∗^*p* *<* 0.05, ^∗∗∗^*p* < 0.0001, ns, not significant, repeated measures two-way ANOVA followed by *post hoc* Bonferroni’s test in **(A,C)** and unpaired student’s *t*-test in **(B,D)**. Error bars represent SEM. EZMT; elevated zero maze test, OFT; open field test.

**FIGURE 3:**
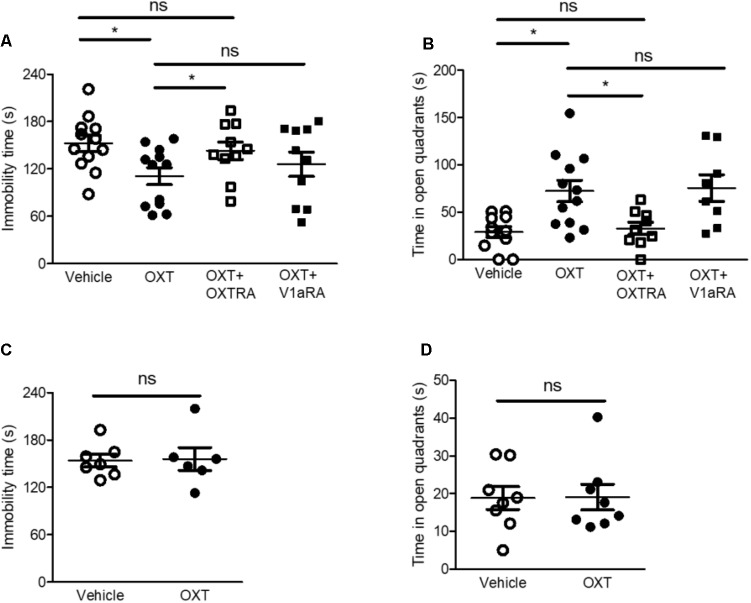
Intra-CeA injection of OXT, but not ICV injection, attenuated isolation-induced depression and anxiety-related behaviors via OXTR in the FST and EZMT, respectively. **(A)** Immobility time of isolated mice after intra-CeA injection of OXT (0.5 μg, *n* = 12), OXT + OXTRA (1 μg, *n* = 10), and OXT + V1aRA (1 μg, *n* = 10) was compared to that after vehicle injection (*n* = 12) in the FST. **(B)** Time spent in the open quadrants by isolated mice after intra-CeA injection of OXT (0.5 μg, *n* = 12), OXT + OXTRA (1 μg, *n* = 9), and OXT + V1aRA (1 μg, *n* = 8) was compared to that after vehicle injection (*n* = 10) in the EZMT. **(C)** Immobility time of isolated mice after ICV injection of OXT (0.5 μg, *n* = 6) was compared to that after vehicle injection (*n* = 7) in the FST. **(D)** Time spent in the open quadrants by the isolated mice after ICV injection of OXT (0.5 μg, *n* = 8) was compared to that after vehicle injection (*n* = 8) in the EZMT. ^∗^*p* < 0.05, ns, not significant, one-way ANOVA followed by *post hoc* Bonferroni’s test in **(A,B)** and unpaired student’s *t*-test in **(C,D)**. Error bars represent SEM. CeA, central amygdala; OXT, oxytocin; OXTR, oxytocin receptor; V1aRA, vasopressin-1a receptor antagonist; OXTRA, oxytocin receptor antagonist; ICV, intracerebroventricular.

Data are expressed as mean ± SEM. All data were analyzed using unpaired *t*-tests, except for the data in **Figures 1A,C**, **2A,C**, **3A,B**, **6**. Data in **Figures 1A,C**, **2A,C** were analyzed using repeated measures two-way ANOVA, and *post hoc* analysis was performed using Bonferroni’s test. Data in **Figures 3A,B** were analyzed using one-way ANOVA, and *post hoc* analysis was performed using Bonferroni’s test. Data in **Figure 6** were analyzed using a paired *t*-test. *p* < 0.05 was considered significant.

## Results

### Long-Term Isolation Induced Depression-Related Behaviors in Male Mice

To investigate whether isolation induced depression in mice, we measured depression-related behaviors using the FST and the SPT every week for 5 weeks. For the first 3 weeks, isolation did not significantly increase depression-related behaviors such as learned helplessness and anhedonia in isolated mice (**Figures 1A,C**). However, after 4 weeks of isolation, depression-related behaviors began to develop in isolated mice compared to group-housed mice as measured by both the FST and the SPT (**Figures 1A,C**). Given that repeated tests influenced behavioral outcomes (**Figure 1A**) and neural circuits (Takao et al., 1995; Badowska-Szalewska et al., 2010) for depression, we measured depression-related behaviors for the first time after the 5-week isolation in another group of mice. In both the FST and the SPT, depression-related behaviors significantly increased in isolated mice compared to group-housed mice after the 5-week isolation (**Figures 1B,D**). These results demonstrated that 5 weeks of isolation induced depression-related behaviors in male mice.

### Long-Term Isolation Elicited Anxiety-Related Behaviors in Male Mice

To examine whether isolation induced anxiety, we measured anxiety-related behaviors using the EZMT and the OFT every week for 5 weeks. In the EZMT and the OFT, for the first 2 weeks, isolation did not significantly elevate anxiety-related behavior as measured by the time spent in the open quadrants and center, respectively (**Figures 2A,C**). However, after 3 weeks of isolation, anxiety-related behaviors started to develop in isolated mice compared to group-housed mice as measured by both the EZMT and the OFT (**Figures 2A,C**). Since repeated tests altered the behavioral results for the measurement of anxiety in group-housed mice (**Figures 2A,C**), we measured anxiety-related behaviors for the first time after the 5-week isolation in another group of mice. In both the EZMT and the OFT, after the 5-week isolation, anxiety-related behaviors significantly increased in the isolated mice compared to group-housed mice (**Figures 2B,D** and **Supplementary Figure S1**). These results showed that 5 weeks of isolation induced anxiety-related behaviors in male mice as well.

### OXT Administration Into the CeA, but Not Intracerebroventricular Injection, Ameliorated Isolation-Induced Depression and Anxiety-Related Behaviors via OXTR

The amygdala plays a critical role in the pathophysiology of anxiety disorders and is associated with the development of subtype of depressive disorders (Slattery and Neumann, 2010b; Neumann and Slattery, 2016). OXT has been reported to reduce anxiety-related behaviors (Neumann and Slattery, 2016). We therefore explored whether intra-CeA injection of OXT reduced isolation-induced depression and anxiety-related behaviors in isolated mice. Intra-CeA administration of OXT (0.5 μg) attenuated isolation-induced depression and anxiety-related behaviors in the FST and the EZMT, respectively (**Figures 3A,B**). Since OXT binds to several receptors such as vasopressin receptors as well as OXTR, and both OXTR and vasopressin-1a receptor (V1aR) are known to modulate social and emotional behaviors (Meyer-Lindenberg et al., 2011; Benarroch, 2013), we used OXTRA and V1aRA to determine the specific receptor that mediated OXT-induced antidepressant and anxiolytic effects. In the FST and the EZMT, co-administration of OXT (0.5 μg) with OXTRA (1 μg) into the CeA did not rescue isolation-induced depression and anxiety-related behaviors in the FST and the EZMT, respectively (**Figures 3A,B**). However, in both tests, co-administration of OXT (0.5 μg) with V1aRA (1 μg) attenuated isolation-induced depression and anxiety-related behaviors (**Figures 3A,B**). We then administered the same dose OXT (0.5 μg) into the lateral ventricles to assess whether OXT acted on the CeA to exert its antidepressant and anxiolytic effects. The same dose of intracerebroventricular (ICV) injection of OXT did not ameliorate isolation-induced depression and anxiety-related behaviors (**Figures 3C,D**). These findings implied that OXT acted on the CeA as an antidepressant and anxiolytic via OXTR.

### OXTR but Not V1aR mRNA Transcription in the CeA Was Reduced After the 5-Week Isolation

**FIGURE 4:**
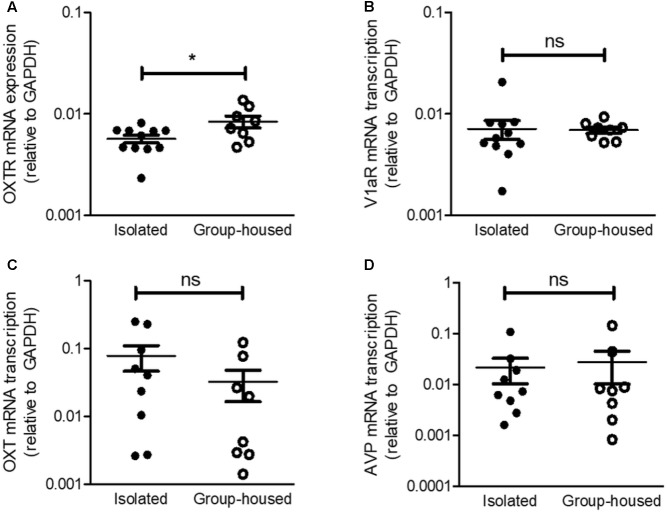
Relative mRNA transcription of OXTR in the CeA, but not that of V1aR, was decreased in isolated mice. **(A)** Comparison of OXTR mRNA transcription between isolated (*n* = 11) and group-housed (*n* = 8) mice in the CeA. **(B)** Comparison of V1aR mRNA transcription isolated (*n* = 11) and group-housed (*n* = 8) mice in the CeA. **(C)** Comparison of OXT mRNA transcription between isolated (*n* = 9) and group-housed (*n* = 8) mice in the PVN. **(D)** Comparison of AVP mRNA transcription between isolated (*n* = 9) and group-housed (*n* = 8) mice in the PVN. ^∗^*p* *<* 0.05, ns, not significant, unpaired student’s *t*-test. Error bars represent SEM. OXTR, oxytocin receptor; V1aR, vasopressin-1a receptor; OXT, oxytocin; AVP, vasopressin; GAPDH, glyceraldehyde 3-phosphate dehydrogenase; CeA, central amygdala.

Given that social isolation was reported to modify OXTR and V1aR mRNA transcription in the hypothalamus (Pournajafi-Nazarloo et al., 2013) and OXT acts on the CeA as an antidepressant and anxiolytic (**Figure 3**), we thus examined mRNA transcription of OXT/vasopressin circuits from the PVN to the CeA. OXTR mRNA transcription was significantly down-regulated in the CeA after the 5-week isolation (**Figure 4A**) but V1aR mRNA transcription remained unchanged (**Figure 4B**). These results were consistent with our previous findings that OXT acted on the CeA as an antidepressant and anxiolytic via OXTR, not V1aR. To interrogate the changes in OXT and vasopressin production after long-term isolation, we examined OXT and hypothalamic vasopressin mRNA transcription. No significant differences were observed in OXT and vasopressin mRNA transcription in the PVN between the isolated and group-housed mice (**Figures 4C,D**).

### Long-Term Isolation Reduced Inhibitory Synaptic Transmission in the CeA

**FIGURE 5:**
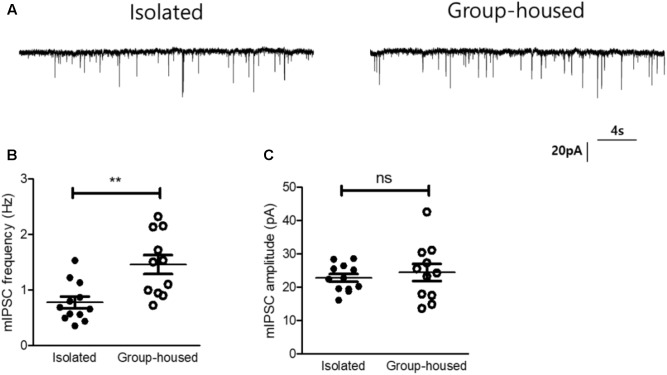
Inhibitory synaptic transmission of the CeA was reduced in isolated mice. **(A)** Representative traces of mIPSCs recorded from the CeA of an isolated mouse (left) and a group-housed mouse (right). **(B)** Comparison of mIPSC frequency between isolated (*n* = 12) and group-housed (*n* = 11) mice. **(C)** Comparison of mIPSC amplitude between isolated (*n* = 12) and group-housed (*n* = 11) mice. ^∗∗^*p* < 0.005, ns, not significant, unpaired student’s *t*-test. Dot represents each cell. Error bars represent SEM. CeA, central amygdala; mIPSC, miniature inhibitory post synaptic currents.

Oxytocin signaling has been known to modulate γ-aminobutyric acid (GABA)-ergic circuits in the lateral part of the CeA and inhibit neurons in the medial part of the CeA through the GABAergic projections (Huber et al., 2005; Viviani et al., 2011; Knobloch et al., 2012), We therefore hypothesized that the reduced OXTR mRNA transcription after long-term isolation may result in functional changes in CeA inhibitory signaling. To test this hypothesis, we examined the effects of long-term isolation on inhibitory synaptic transmission in the medial part of the CeA (CeM). In studying the inhibitory synaptic transmission, we blocked all action potential-driven synaptic events by including tetrodotoxin (TTX) in the slice perfusion medium. This experimental strategy allowed us to focus on mIPSC, the changes in the frequency and amplitude of which would indicate changes in synaptic transmission originating pre- and post-synaptically. Our results demonstrated that mIPSC frequency in the CeM was significantly decreased in isolated mice (0.77 ± 0.1 Hz, *n* = 12) compared to group-housed mice (1.47 ± 0.17 Hz, *n* = 11) (**Figures 5A,B**). However, the mIPSC amplitude in the CeM was not significantly changed after long-term isolation (isolated mice, 22.7 ± 1.1 pA, *n* = 12; group-housed mice, 24.4 ± 2.5 pA, *n* = 11) (**Figures 5A,C**). These results suggested that long-term isolation attenuated inhibitory synaptic transmission in the CeM via reduction of GABA release from presynaptic terminals.

### OXT Rescued the Decreased Local GABAergic Synaptic Transmission in the CeA of Isolated Mice via OXTR

**FIGURE 6:**
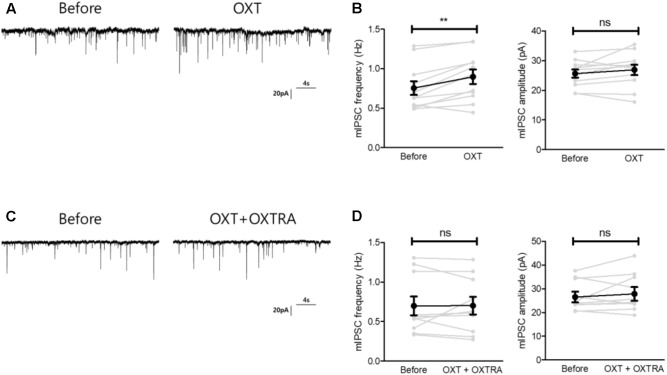
Oxytocin treatment restored the reduced inhibitory synaptic transmission of the CeA of isolated mice via OXTR. **(A)** Representative traces of mIPSCs recorded from the CeA of isolated mice before treatment (left) and 5 min after OXT treatment (right). **(B)** Comparison of mIPSC frequency (left) and amplitude (right) between pre- and post-treatment with OXT in isolated mice (*n* = 11). **(C)** Representative traces of mIPSCs recorded from the CeA of isolated mice before treatment (left) and 5 min after OXT + OXTRA treatment (right). **(D)** Comparison of mIPSC frequency (left) and amplitude (right) between pre- and post-treatment with OXT + OXTRA in isolated mice (*n* = 11). ^∗∗^*p* < 0.005, ns, not significant, paired *t*-test. Dot represents each cell. Error bars represent SEM. OXT, oxytocin; CeA, central amygdala; OXTR, oxytocin receptor; mIPSC, miniature inhibitory post synaptic currents; OXTRA, oxytocin receptor antagonist.

**FIGURE 7:**
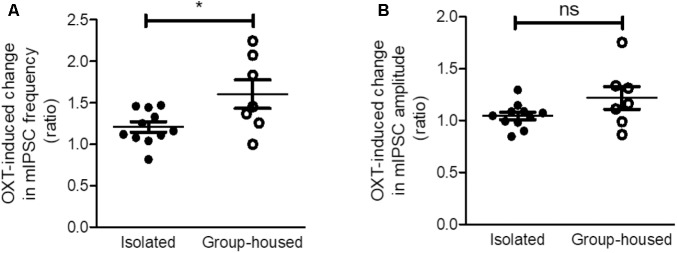
The OXT-induced increase in inhibitory synaptic transmission was greater in group-housed mice than in isolated mice. **(A)** Comparison of the OXT-induced change in mIPSC frequency between isolated (*n* = 11) and group-housed (*n* = 7) mice. **(B)** Comparison of the OXT-induced change in mIPSC amplitude between isolated (*n* = 11) and group-housed (*n* = 7) mice. ^∗^*p* < 0.05, ns; not significant, unpaired student’s *t*-test. Dot represents each cell. Error bars represent SEM. OXT, oxytocin; mIPSC, miniature inhibitory post synaptic current.

As OXT administration rescued isolation-induced depression and anxiety-related behaviors (**Figure 3**), we investigated whether OXT treatment restored the isolation-induced decrement of CeM inhibitory synaptic transmission. Analysis of mIPSC recordings from isolated mice showed that OXT increased the mIPSC frequency (**Figure 6A**) from 0.75 ± 0.08 to 0.89 ± 0.09 Hz (*n* = 11) in the CeM but did not alter the mIPSC amplitude (**Figure 6B**, from 25.6 ± 1.3 to 26.9 ± 1.7 Hz, *n* = 11). To interrogate the effects of down-regulated OXTR mRNA transcription on possible functional changes in CeM inhibitory synaptic transmission, we compared the extent of OXT-induced changes between isolated and group-housed mice. The increase in the mIPSC frequency after OXT treatment, but not in the mIPSC amplitude, was greater in group-housed mice than in isolated mice (**Figure 7A,B**). We also observed that co-application of OXT with OXTRA blocked the OXT-induced increase in the mIPSC frequency in the CeM of isolated mice (**Figures 6C,D**). Taken together, these results suggested that in accordance with the behavioral changes, OXT treatment restored the isolation-induced decrement of inhibitory synaptic transmission in the CeM via OXTR. Furthermore, the decrease in inhibitory synaptic transmission after long-term isolation could result in part from the reduction of OXTR that mediates the excitatory effect of OXT on the inhibitory GABAergic neurons innervating the cells.

## Discussion

The main findings of this study are: (1) that long-term isolation down-regulated mRNA transcription of OXTR in the CeA, (2) that long-term isolation reduced GABAergic synaptic transmission in the CeA, and (3) that OXT treatment restored the functional changes in inhibitory synaptic transmission in the CeA and also rescued the behavioral deficits in the FST and the EZMT produced by long-term isolation via OXTR.

Here, we revealed that mice showed significantly increased depression and anxiety-related behaviors after more than 4 weeks of isolation (**Figures 1**, **2**). These findings agree with previous studies showing that social isolation affected depression and anxiety-related behaviors. A week of isolation increased anxiety-related behaviors, but did not affect depression-related behaviors (Kwak et al., 2009). After isolation followed by an enriched environment, isolated mice developed anxiety-related behaviors in the OFT, but not depression-related behaviors in the learned-helplessness paradigm (Chourbaji et al., 2005). The discrepancy between previous studies and our data may be attributed to the duration of isolation and the enriched environment, as prolonged isolation with impoverished circumstances was reported to elicit both depression- and anxiety-related behaviors (Ieraci et al., 2016).

Oxytocin has been known to act as an anxiolytic (Neumann and Slattery, 2016) and the CeA has been implicated in anxiety and fear responses (Ciocchi et al., 2010; Knobloch et al., 2012), but the loci of the anxiolytic action of OXT in the context of social isolation remain unclear. Moreover, a few human studies have indicated that OXT has anti-depressant properties (Scantamburlo et al., 2011; Cochran et al., 2013). We aimed to determine whether OXT was involved with isolation-induced behavioral abnormalities such as depression and anxiety-related behaviors and whether OXT acts on the CeA that contains abundant expression of OXTR (Yoshida et al., 2009; Boccia et al., 2013). We therefore compared the brain site-specific effects of OXT on isolation-induced abnormal behaviors. Consistent with previous studies (Slattery and Neumann, 2010a; Zoicas et al., 2014), ICV injection of OXT did not affect isolation-induced abnormal psychiatric behaviors. However, administration of the same dose of OXT into the CeA or ICV injection of a dose 10 times higher (5 μg) (data not shown) attenuated isolation-induced behavioral deficits. Since OXT binds to vasopressin receptors as well as OXTR (Manning et al., 2012), we administered specific receptor antagonists to identify which receptor mediated the anxiolytic and antidepressant effects of OXT. Consistent with previous studies demonstrating that OXTR is more related to the relief of social stress than vasopressin receptors (Benarroch, 2013), we confirmed that OXT exerted its anti-depressant and anxiolytic effects via OXTR, not V1aR. Taken together, these findings suggested that OXT acted specifically on the CeA via OXTR to modulate depression and anxiety-related behaviors. It is possible that the lack of effects of OXT in human studies is due to the insufficient delivery of OXT into the CeA.

Expression and transcription of OXTR is under more complicating regulation than other G-protein coupled receptors (Zingg and Laporte, 2003). For example, prolonged stimulation of OXTR by an agonist induces OXTR down-regulation (Gimpl and Fahrenholz, 2001). Social isolation was reported to be associated with down-regulation of hypothalamic OXTR mRNA transcription (Pournajafi-Nazarloo et al., 2013). Moreover, deprivation of social interactions and chronic stress produced by isolation are involved with not only decrease but also increase in the secretion of OXT (Pournajafi-Nazarloo et al., 2013; Han et al., 2016b). Thus, our observation that the mRNA transcription of OXTR, but not that of V1aRA, was reduced in the CeA after the 5-week isolation (**Figures 4A,B**) may be attributed to two possible scenarios. First, the altered secretion of OXT after long-term isolation possibly decreased OXTR mRNA transcription in the CeA. Alternatively, social isolation might influence the transcription of OXTR mRNA regardless of the change in OXT secretion.

Oxytocinergic circuits from the hypothalamus have been known to modulate GABA signaling in the CeA (Huber et al., 2005; Viviani et al., 2011; Knobloch et al., 2012) and decreases in OXTR expression modulate presynaptic GABA release (Ripamonti et al., 2017). In the current study, we found that OXTR mRNA in the CeA is down-regulated after long-term isolation and OXT rescues the isolation-induced depression- and anxiety-related behaviors by acting on OXTR in the CeA. To test the hypothesis that the alteration of GABAergic transmission in CeA neurons resulting from OXTR down-regulation underlies the emergence of the isolation-induced depression- and anxiety-related behaviors, we performed whole-cell patch-clamp recordings in acutely prepared brain slices from isolated and group-housed mice. From these experiments, we learned that mIPSCs in CeA neurons were lower in frequency in the case of isolated, than group-housed, mice and that OXT, by acting through OXTR, increased the frequency of mIPSC more robustly in the group-house mice. These results indicate that long-term isolation may elicit the depression- and anxiety-related behaviors by down-regulating the OXTR, which is excitatory to the inhibitory GABAergic neurons innervating CeA neurons, and hence disinhibiting these cells. However, we could not exclude the possibility that other than OXT, signaling molecules such as serotonin and catecholamine could also contribute to isolation-induced circuit plasticity in the CeA (Karkhanis et al., 2015; Yu et al., 2018).

Our data from electrophysiological experiments partly disagree with the findings of Huber et al. (2005). The IPSCs of CeA cells recorded in the absence of TTX by these authors were much greater in amplitude and frequency than the ones detected in our experiments. Moreover, the increase in IPSC frequency induced by the application of OXT was much more robust in Huber et al. (2005) experiments. Apparently, these discrepancies might be due to the fact that Huber et al. (2005) recorded spontaneous IPSCs, whereas we obtained mIPSCs after the blockade of all action potential-dependent synaptic events with TTX. In this context, it is interesting to note that even in the presence of TTX, OXTR activation can depolarize the membrane potential, thus leading to action potential-independent GABA release (Cohen et al., 1992; Murai et al., 1998; Harden and Frazier, 2016).

## Conclusion

In conclusion, our data reveal a neural mechanism underlying isolation-induced depressive and anxiety behaviors. We propose that the decrease in OXTR mRNA transcription after long-term isolation may induce depressive and anxiety disorders by reducing GABA release and consequently, disinhibiting CeA neurons. These results imply that modulation of OXTR signaling and expression in the CeA is a promising target for developing treatments for depressive and anxiety disorders.

## Author Contributions

RH and Y-BK equally contributed to this manuscript. HN and YK conceived the idea, obtained funding for the study, and guided the project. RH performed the behavioral tests and stereotaxic injection and Y-BK performed whole-cell path clamp. E-HP performed and analyzed stereotaxic injection and behavioral results. JK performed behavioral tests. CR performed and analyzed electrophysiology results. HYK and JL performed and analyzed qPCR. KP and CS performed stereotaxic and analyzed electrophysiology data, respectively. HK and SB conceived the idea and analyzed the results. YK conceived the idea. RH, Y-BK, HJK, and HN wrote the manuscript. All authors gave final approval and agree to be accountable for all aspects of the work.

## Conflict of Interest Statement

The authors declare that the research was conducted in the absence of any commercial or financial relationships that could be construed as a potential conflict of interest.

## Supplementary Material

FIGURE S1 Isolated mice showed anxiety-related behaviors without hyperactivity in the OFT and anxiety-related behaviors in the EZMT after 5-week isolation. **(A)** Comparison of the number of center visits between isolated (*n* = 12) and group-housed mice (*n* = 12) in the OFT after the 5-week isolation. **(B)** Comparison of the total movement distance between isolated (*n* = 12) and group-housed mice (*n* = 12) in the OFT after the 5-week isolation. **(C)** Comparison of the number of head dips between isolated (*n* = 12) and group-housed mice (*n* = 12) in the EZMT after the 5-week isolation. ^∗^*p* < 0.05, ^∗∗∗^*p* < 0.001, ns, not significant, unpaired student’s *t*-test. Error bars represent SEM.
