# Supplementary figures and images for: Long-Term Isolation Elicits Depression and Anxiety-Related Behaviors by Reducing Oxytocin-Induced GABAergic Transmission in Central Amygdala
Source: Front Mol Neurosci. 2018 Aug 14;11:246. doi: 10.3389/fnmol.2018.00246 (PMC6104450; doi:10.3389/fnmol.2018.00246)

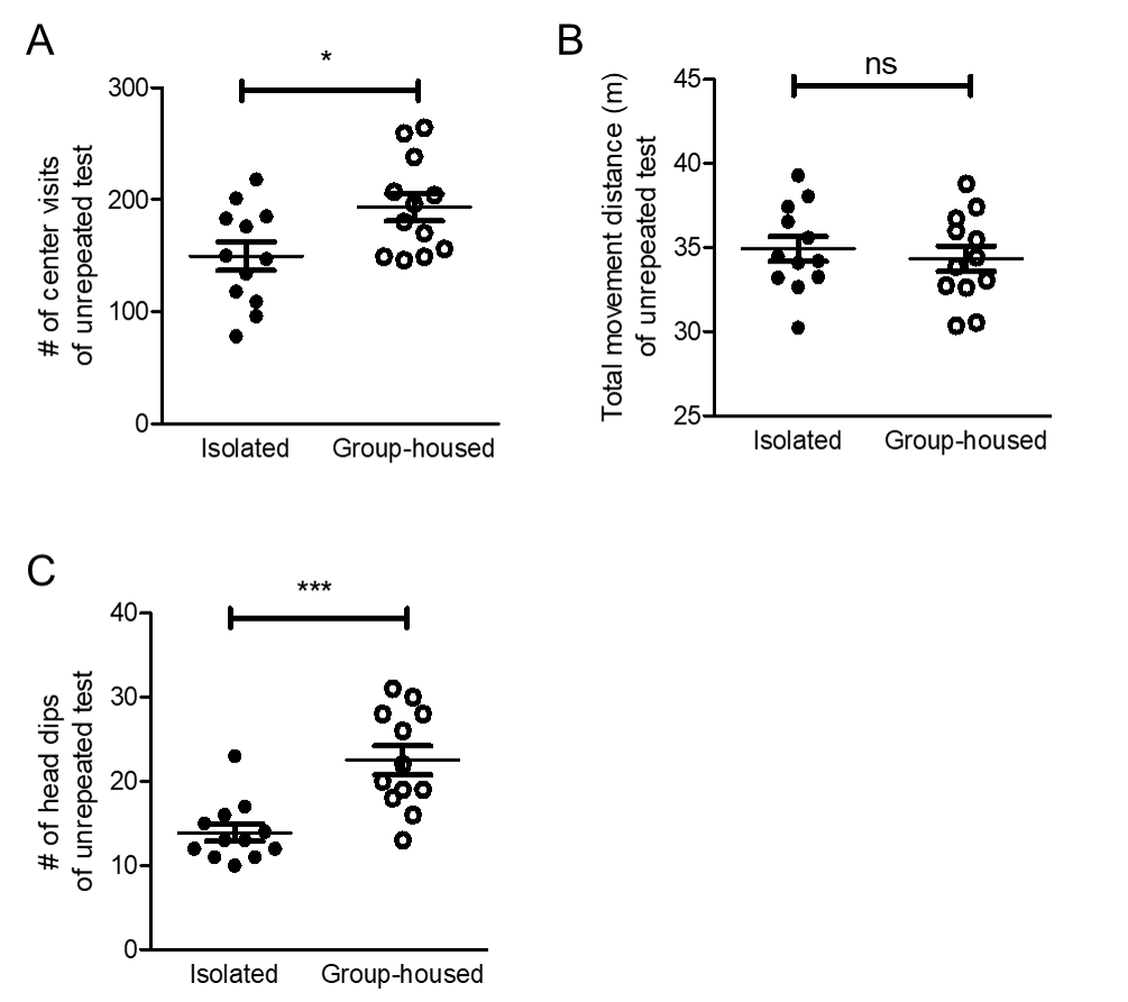

Supplement: FIGURE S1 — Isolated mice showed anxiety-related behaviors without hyperactivity in the OFT and anxiety-related behaviors in the EZMT after 5-week isolation. (A) Comparison of the number of center visits between isolated (n = 12) and group-housed mice (n = 12) in the OFT after the 5-week isolation. (B) Comparison of the total movement distance between isolated (n = 12) and group-housed mice (n = 12) in the OFT after the 5-week isolation. (C) Comparison of the number of head dips between isolated (n = 12) and group-housed mice (n = 12) in the EZMT after the 5-week isolation. ∗p < 0.05, ∗∗∗p < 0.001, ns, not significant, unpaired student’s t-test. Error bars represent SEM. [file Image_1.TIF]
